# Supplementary material for: Biophysical ordering transitions underlie genome 3D re-organization during cricket spermiogenesis
Source: Nat Commun. 2023 Jul 13;14:4187. doi: 10.1038/s41467-023-39908-1 (PMC10345107; doi:10.1038/s41467-023-39908-1)
Supplement: Supplementary file 1 — Supplementary Information [file 41467_2023_39908_MOESM1_ESM.pdf]

Supplementary Figure 1

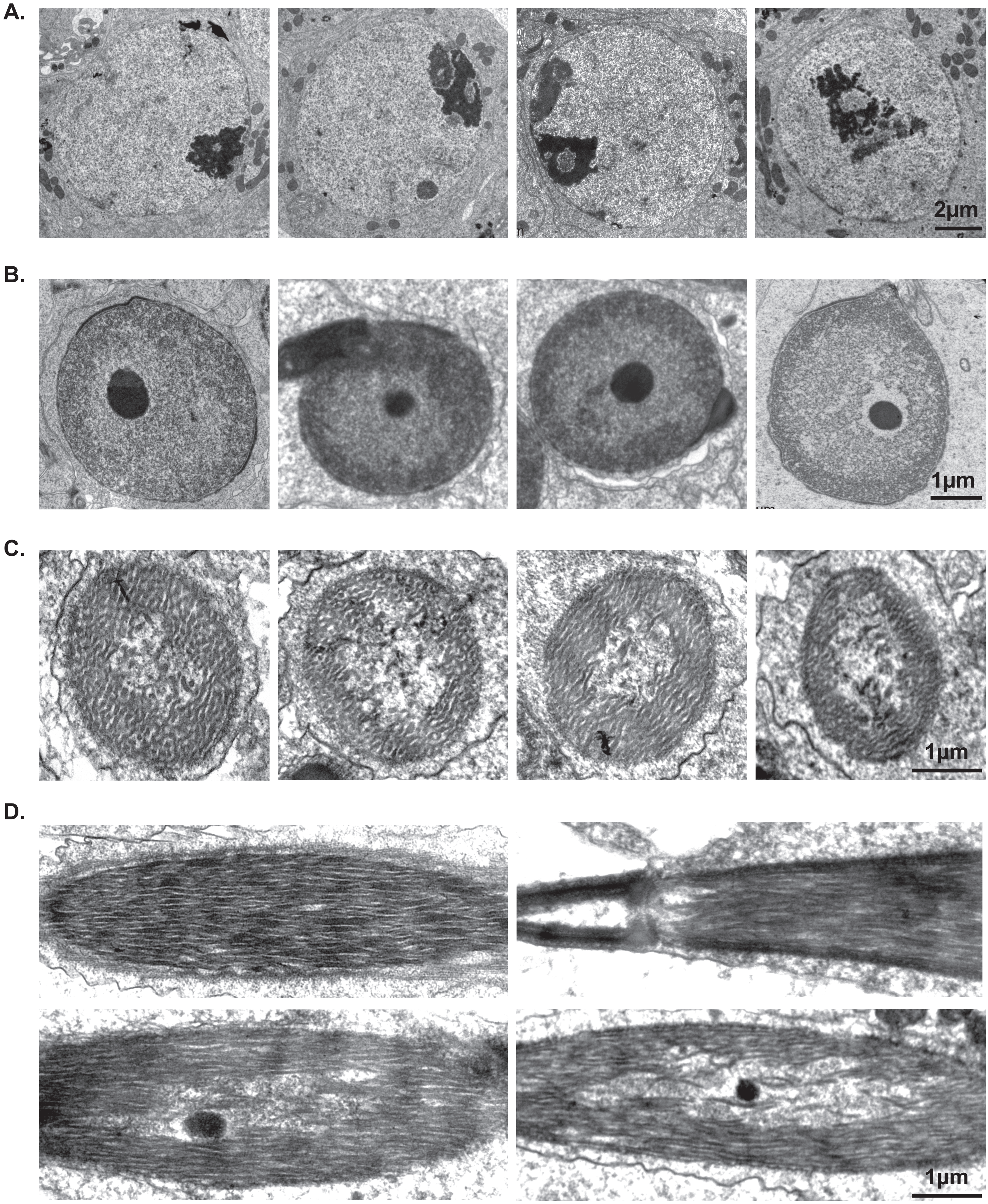

Supplementary Figure 1: Additional representative electron micrographs used for density and orientational quantifications in (A) Spermatocytes. **(B)** Round spermatids. **(C)** Elongating spermatids (transversal sections). **(D)** Elongating spermatids (longitudinal sections).

Supplementary Figure 2

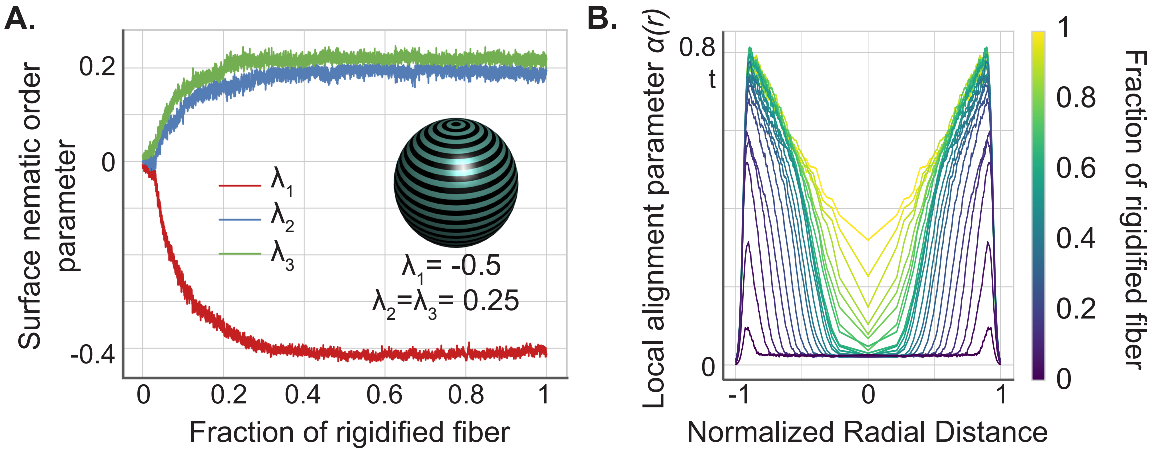

Supplementary Figure 2: Additional ordering parameters in nucleated rigidification simulations. (A) Quantification of the surface-oriented spool in terms of the eigenvalues of the surface nematic Q-tensor, which evidence a transition from a disordered state (λ_1=λ_2=λ_3=0) to a coaxial, torus-like structure (λ_1=-0.5, λ_2=λ_3=0.25) as the rigidification of the fiber progresses. Note the rapid convergence towards the spooled state as the fraction of the rigidified fiber exceeds ~30%. **(B)** Corresponding radial fiber alignment profiles (see Methods). The discretized local alignment parameter α displays marked peaks of increasing width near the nuclear envelope walls (r=±R), characteristic of orientational wetting, in which nematic order is nucleated at the nuclear periphery and gradually propagates through the entire cavity as rigidification progresses.

Supplementary Figure 3

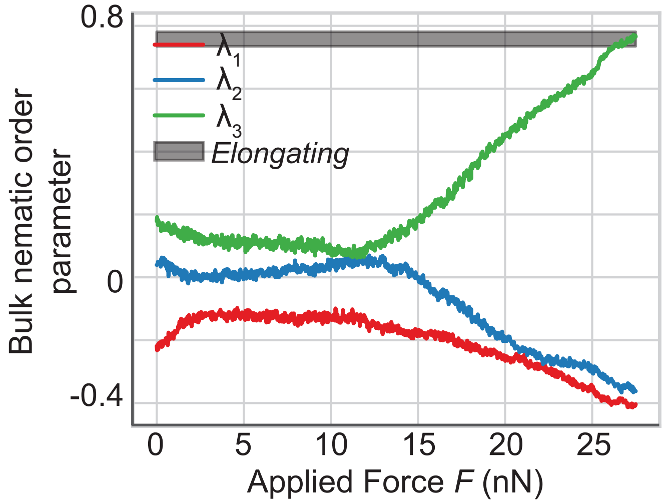

Supplementary Figure 3: Additional ordering parameters in nuclear stretching simulations. Eigenvalues of the bulk nematic Q-tensor, evidencing a transition from a spool-like oblate structure ($\lambda_3 \cong \lambda_2 \gg \lambda_1$) to a prolate nematic arrangement ($\lambda_1 \cong \lambda_2 \ll \lambda_3$) characterized by the strong alignment of the fiber along the direction of nuclear stretching. In this regime, which increasingly prevails as the applied force is raised beyond the approximate threshold $F \gtrsim 15$nN, the alignment parameter α simply reduces to the largest eigenvalue λ_3 of the nematic Q-tensor (Fig.5E), which is more traditionally used as a simple measure of uniform, uniaxial orientational order.
