# Supplementary material for: Biophysical ordering transitions underlie genome 3D re-organization during cricket spermiogenesis
Source: Nat Commun. 2023 Jul 13;14:4187. doi: 10.1038/s41467-023-39908-1 (PMC10345107; doi:10.1038/s41467-023-39908-1)
Supplement: Supplementary file 3 — Reporting Summary [file 41467_2023_39908_MOESM3_ESM.pdf]

nature portfolio

Corresponding author(s): Guillermo A. Orsi, Daniel Jost, Benjamin Loppin

Last updated by author(s): Apr 24, 2023

Reporting Summary

Nature Portfolio wishes to improve the reproducibility of the work that we publish. This form provides structure for consistency and transparency in reporting. For further information on Nature Portfolio policies, see our Editorial Policies and the Editorial Policy Checklist.

Statistics

For all statistical analyses, confirm that the following items are present in the figure legend, table legend, main text, or Methods section.

n/a	Confirmed	
☐	☒	The exact sample size (*n*) for each experimental group/condition, given as a discrete number and unit of measurement
☐	☒	A statement on whether measurements were taken from distinct samples or whether the same sample was measured repeatedly
☐	☒	The statistical test(s) used AND whether they are one- or two-sided<br>*Only common tests should be described solely by name; describe more complex techniques in the Methods section.*
☒	☐	A description of all covariates tested
☒	☐	A description of any assumptions or corrections, such as tests of normality and adjustment for multiple comparisons
☐	☒	A full description of the statistical parameters including central tendency (e.g. means) or other basic estimates (e.g. regression coefficient) AND variation (e.g. standard deviation) or associated estimates of uncertainty (e.g. confidence intervals)
☒	☐	For null hypothesis testing, the test statistic (e.g. *F*, *t*, *r*) with confidence intervals, effect sizes, degrees of freedom and *P* value noted<br>*Give P values as exact values whenever suitable.*
☒	☐	For Bayesian analysis, information on the choice of priors and Markov chain Monte Carlo settings
☒	☐	For hierarchical and complex designs, identification of the appropriate level for tests and full reporting of outcomes
☒	☐	Estimates of effect sizes (e.g. Cohen's *d*, Pearson's *r*), indicating how they were calculated

Our web collection on statistics for biologists contains articles on many of the points above.

Software and code

Policy information about availability of computer code

Data collection	No software was used for data collection
Data analysis	Images were analyzed with Zen imaging software 3.0 (Carl Zeiss) and FIJI software 2.7.0. Polymer simulations and analysis were performed with custom code available at GitHub: https://github.com/mtortora/HOOMD-Nucl.git

For manuscripts utilizing custom algorithms or software that are central to the research but not yet described in published literature, software must be made available to editors and reviewers. We strongly encourage code deposition in a community repository (e.g. GitHub). See the Nature Portfolio guidelines for submitting code & software for further information.

Data

Policy information about availability of data

All manuscripts must include a data availability statement. This statement should provide the following information, where applicable:

- Accession codes, unique identifiers, or web links for publicly available datasets
- A description of any restrictions on data availability
- For clinical datasets or third party data, please ensure that the statement adheres to our policy

The imaging data generated in this study and used for analysis have been deposited in the Zenodo database under accession code https://doi.org/10.5281/zenodo.8043115

Human research participants

Policy information about studies involving human research participants and Sex and Gender in Research.

Reporting on sex and gender	N/A
Population characteristics	N/A
Recruitment	N/A
Ethics oversight	N/A

Note that full information on the approval of the study protocol must also be provided in the manuscript.

Field-specific reporting

Please select the one below that is the best fit for your research. If you are not sure, read the appropriate sections before making your selection.

☒ Life sciences ☐ Behavioural & social sciences ☐ Ecological, evolutionary & environmental sciences

For a reference copy of the document with all sections, see nature.com/documents/nr-reporting-summary-flat.pdf

Life sciences study design

All studies must disclose on these points even when the disclosure is negative.

Sample size	All groups of experimental data used for quantifications included 10 or more samples. We determined this to be sufficient owing to low variability between samples, as reflected in small standard errors shown in Figure 2F, 3D, and 5E.
Data exclusions	No data was excluded.
Replication	All experiments were reproduced at least 3 times. No discordant results were found.
Randomization	Our study did not involve separating samples into groups.
Blinding	Our study did not involve comparing conditions that would require blinding.

Reporting for specific materials, systems and methods

We require information from authors about some types of materials, experimental systems and methods used in many studies. Here, indicate whether each material, system or method listed is relevant to your study. If you are not sure if a list item applies to your research, read the appropriate section before selecting a response.

Materials & experimental systems

n/a	Involved in the study	
☐	☒	Antibodies
☒	☐	Eukaryotic cell lines
☒	☐	Palaeontology and archaeology
☐	☒	Animals and other organisms
☒	☐	Clinical data
☒	☐	Dual use research of concern

Methods

n/a	Involved in the study	
☒	☐	ChIP-seq
☒	☐	Flow cytometry
☒	☐	MRI-based neuroimaging

Antibodies

Antibodies used	anti-H3 antibody (abcam #1791), anti-H4ac (Merck Millipore #06-598), anti-Histones (Millipore #MABE71, clone F152.C25.WJJ), anti-αTubulin (Sigma #T9026), HRP-coupled secondary (Agilent Dako #P044801-2).
Validation	The anti-H3 antibody was used for Western blots. The anti-H4ac, anti-Histones, anti-αTubulin were used for immunofluorescence. Due to high conservation of the targeted proteins, these antibodies show broad reactivity across species including Drosophila and vertebrates, and have been validated for WB and IF, as indicated by the manufacturers, supporting their use in crickets. Our results

further validate their use in crickets as expected protein sizes (in WB) and sub-cellular distributions (in IF) were observed in control conditions (see Figures 1A, 1B, 1C, 4 and 5A).

Animals and other research organisms

Policy information about studies involving animals; ARRIVE guidelines recommended for reporting animal research, and Sex and Gender in Research

Laboratory animals	This manuscript was entirely performed on Gryllus bimaculatus, white strain. All individuals were adults (6weeks to 3 months of age).
Wild animals	This study did not involve wild animals.
Reporting on sex	In this study we report observations either on testes (only male individuals) or on sperm samples extracted from speramtheca (only female individuals).
Field-collected samples	No samples were collected in the field.
Ethics oversight	No ethical approval was required for our study, following the current regulations in France for experiments using laboratory insects.

Note that full information on the approval of the study protocol must also be provided in the manuscript.
